# Towards understanding specific ion effects in aqueous media using thermodiffusion

**DOI:** 10.1140/epje/s10189-022-00164-8

**Published:** 2022-02-01

**Authors:** Shilpa Mohanakumar, Simone Wiegand

**Affiliations:** 1 IBI-4:Biomacromolecular Systems and Processes, Forschungszentrum Jülich GmbH, D-52428 Jülich, Germany; 2 Chemistry Department–Physical Chemistry, University Cologne, D-50939 Cologne, Germany

## Abstract

**Abstract:**

Specific ion effects play an important role in scientific and technological processes. According to Hofmeister, the influence on the hydrogen bond network depends on the ion and leads to a specific order of the ions. Also thermodiffusion the mass transport caused by a temperature gradient is very sensitive to changes of the hydrogen bond network leading to a ranking according to hydrophilicity of the salt. Hence, we investigate various salt solutions in order to compare with the Hofmeister concept. We have studied three different sodium salts in water as a function of temperature (25–45$^\circ$C) and concentration (0.5–5 mol kg$^{-1}$) using Thermal Diffusion Forced Rayleigh Scattering (TDFRS). The three anions studied, carbonate, acetate and thiocyanate, span the entire range of the Hofmeister series from hydrophilic to hydrophobic. We compare the results with the recent measurements of the corresponding potassium salts to see to what extent the cation changes the thermodiffusion of the salt.

**Graphic abstract:**

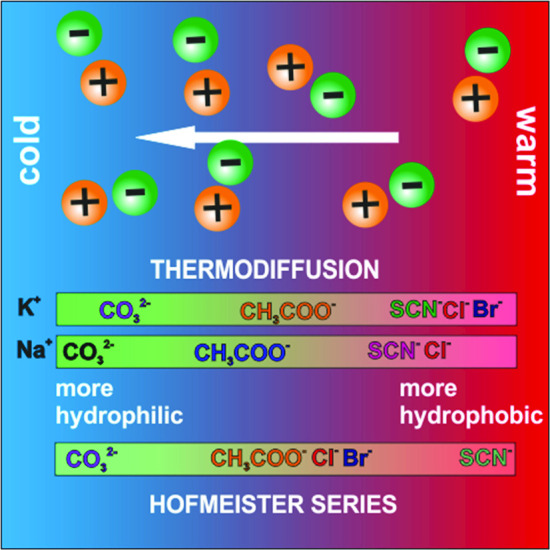

**Supplementary Information:**

The online version contains supplementary material available at 10.1140/epje/s10189-022-00164-8.

## Introduction

In recent years, thermodiffusion or thermophoresis, which is the mass transport caused by a temperature gradient has gained a lot of interest [1–3]. In the steady state, the Soret coefficient $S_\mathrm {T}=D_\mathrm {T}/D$ is defined as the ratio of the thermal diffusion $D_\mathrm {T}$ and the diffusion coefficient *D*. A positive and negative $S_\mathrm {T}$ corresponds to accumulation of the solute molecules in the cold and the warm region, respectively. The established concentration difference $\Delta c=-c(1-c)S_\mathrm {T}\Delta T$ depends also on the applied temperature difference $\Delta T$ and the mass fraction *c*. The research boost in biophysics and bio medicine in recent years can mainly be attributed to the detection of binding reactions via the change in thermophoresis [1, 4]. MicroScale Thermophoresis(MST) gives access to the dissociation constant $K_d$ and molar ratio *n*, but the physical origin for the change in the thermophoretic behavior upon binding is so far microscopically not understood. It is known that during the binding with the ligand structural modifications of the protein occur and additionally the interfacial waters and the hydrogen bond network play an important role [5]. From previous thermophoretic studies of non-ionic compounds it has been revealed that thermodiffusion is strongly correlated with the hydrophilicity of the solute molecules [6].

**Fig. 1:**
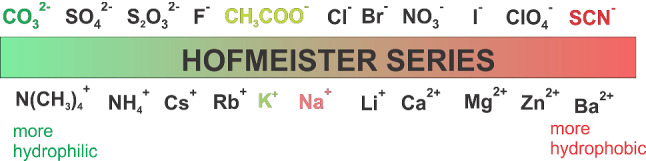
Sketch of the cations and anions of the investigated salts with their probable position according to the Hofmeister series [22, 49]. From left to right, the ions in general becomes more hydrophobic

Specific ion effects are important in numerous fields of science and technology [7, 8]. Since the pioneering work of Hofmeister, it is known that most aqueous physicochemical processes not only depend on ion concentration and valency, but also on the ion type [9–11]. For instance, cells use the ionic selectivity of ion channels to process information through the organism [12]. Different simulation models [13, 14] show a hypothetical variation in temperature at the level of the ion channels, due to the flow of the ions from the inside to the outside, during the genesis of the action potential. Such a non-uniform temperature gradient could then lead to a concentration gradient due to thermodiffusion and influence the signaling. Systematic studies of aqueous potassium salt solutions show that thermodiffusion is also sensitive to the specific ions [15]. This study has illustrated that the thermophoretic behavior of the anion correlates with its position in the Hofmeister series [10, 16]. Ion specific effects influence also the stability, solubility, reactivity and function of bio-molecules [17, 18]. It is argued that the water molecules in the hydration layer of the protein and the dynamics of hydrogen bond networks are influenced by the salts effecting the proteins, but the mechanism is not understood on a microscopic level [19]. Hofmeister [9, 20, 21] developed an empirical concept that ranks both cations and anions based on salt-specific effects, especially their ability to salt out proteins [10, 16, 22]. Fig. 1 shows the Hofmeister series for cations and anions as we know it today. Ions at the left end of the series are well hydrated (*hydrophilic*) and are called “*cosmotropic*” (water structure maker). Ions at the right end of the series are poorly hydrated (*hydrophobic*) and are also referred to as “*chaotropes*” (water structure breaker) [23]. These ion-specific effects are greater for anions than for cations. Since ions are known to change the structure and dynamics of water, this also affects the heat of transfer and thus thermal diffusion [24].

For aqueous solutions the *temperature* dependence of $S_\mathrm {T}$ can often be described using an empirical equation proposed by Iacopini and Piazza [25],

1 $$\begin{aligned} {S_{\mathrm{T}}}\left( T \right) = S_{\mathrm{T}}^\infty \left[ {1 - \exp \left( {\frac{{{T^ *} - T}}{{{T_0}}}} \right) } \right] \;. \end{aligned}$$

where $S_{{T}}^\infty$ ,$T^{*}$ and $T_{0}$ are adjustable parameters that refer to the $S_\mathrm {T}$ at infinite temperature, the temperature at which a sign change of $S_\mathrm {T}$ occurs, and a parameter to describe the curvature, respectively. The temperature dependence of $S_\mathrm {T}$ flattens with increasing temperature indicating that fewer hydrogen bonds can break. Eq. 1 describes the temperature dependence of many diluted non-ionic solutes in water [3, 26], but fails for others like ethanol in water [27]. However, while $S_\mathrm {T}$ of ionic solutes can be described for all concentrations by Eq. 1 this is not the case for salts with larger organic side groups at low and for non-ionic solutes at higher concentrations [6, 15, 28, 29].

To describe the *temperature* and *concentration* dependence the following empirical Ansatz suggested by Wittko and Köhler [30] can be used,

2 $$\begin{aligned} S_\mathrm {T}(m,T)=\alpha (m)\beta (T)+S_\mathrm {T}^i \end{aligned}$$

with polynomial serial expansions for $\alpha (m)$ and $\beta (T)$

3 $$\begin{aligned} \begin{array}{l} \alpha (m) = {a_0} + {a_1}m + {a_2}{m^2} + {a_3}{m^3} + \ldots ,\\ \beta (T) = 1 + {b_1}\left( {T - {T_0}} \right) + {b_2}{\left( {T - {T_0}} \right) ^2} + \ldots . \end{array} \end{aligned}$$

*m* is the molality, $T_0$ is an arbitrary reference temperature, set to $T_0=25^\circ$C and $S_\mathrm {T}^i$ is a temperature and concentration independent constant. Although the approach was originally developed for non-polar systems it can also been used to describe the temperature and concentration dependence of polar aqueous solutions [6, 15]. In our recent study of polar systems we observed a correlation between $S_\mathrm {T}^i$ and $\log P$ [15]. The partition coefficient *P* is a measure for the relative difference of solubility for a solute in two different solvents. Most commonly used is the octanol/water partition coefficient, because it is used for modeling physiological and environmental transport processes and an important parameter for drug compounds [31, 32]. In a system where a solute can diffuse freely between two phases, *P* is the ratio of its equilibrium concentration in octanol over that in water, so a negative $\log P$ signifies stronger hydrophilicity. Further $\log P$ of a given solute molecule is proportional to its activity coefficient in water $\log \gamma _{water}$ and is used as a measure of solute-solvent interactions in aqueous solutions [33].

In order to get a physical picture of the thermodiffusion of salt solutions, we also need to identify the diffusing entity. With increasing concentration, ion pairs and larger clusters can form in solution. This implies different entities respond to the applied temperature gradient and the solutions might be inhomogenous containing different entities [15, 29, 34, 35]. There are several theoretical models to describe the concentration dependence of the experimentally determined diffusion coefficient *D* in electrolyte solutions [36–38]. The theories which provide an accurate fit to the experimental data are generally valid only at dilute concentrations since they are based on Debye-Hückel ion atmosphere model that includes electrophoretic effects [39–41]. Ions are in general moving under the influence of two forces:

1. a gradient of the chemical potential, which is the main contribution to the movement of ions
2. an electric field produced by the motion of oppositely charged ions

Based on the early work by Nernst [36] and later by Onsager and Fuoss [37] *D* can be described as follows:

4 $$\begin{aligned} D = (D_0+\Delta _n)(1+c(\frac{d \ln \gamma _\pm }{dc})) \end{aligned}$$

where $D_0$ is the Nernst limiting value of the diffusion coefficient, *c* is the concentration of solute in moles per volume and $\gamma _\pm$ is the mean molar activity coefficient of the salt. $\Delta _n$ is the contribution from the electrophoretic term which was introduced by Onsager and Fuoss [37] into the Nernst-Hartley equation [36]. This theoretical approach predicts a minimum in *D* at low concentrations and then a constant increase at high concentrations. To achieve a good description of the experimental data an effective cationic diameter is used to account for the hydration layer [42]. The theoretical approach fails to describe a drop of the diffusion coefficient due to aggregation as observed for example for potassium thiocyanate (KSCN) [15, 19, 35, 43].

The change of the diffusing entity with concentration has also been observed in computer simulations of aqueous solution of LiCl [34]. They find single ion diffusion at low concentrations $c<$0.1 mol/l, ion pairs at intermediate concentrations between 0.1 and 1 mol/L and ion clouds with counter ions at high concentrations $c>$1 mol/L. Simulations carried out in aqueous solutions of potassium and sodium salts depicted two scenarios of cluster formation [19, 44–46]. In some salt solutions closely packed ion clusters are formed with increasing concentration, while others build spatially extended ion networks similar to the water network exhibiting an exceptionally high solubility limit in liquid water. For many salts the ion structures do not change, when the cation is exchanged, but for some systems a change from ion cluster formation to network-like structures is observed. Another molecular dynamic study investigated the cation influence by comparing potassium and sodium acetate in aqueous solution [47]. Their study revealed that the association constant to form ion pairs is higher in case of the cation Na$^{+}$ compared K$^{+}$. In general it is expected that hydration of simple anions are quite different, both structurally and dynamically, from hydration of cations, so that a single concept of water structure will not suffice to characterize it [48].

In the previous study the anions of the salts studied covered the entire range of the Hofmeister series, while the cation potassium was always the same [15] . In this work we want to explore the specific effects of cations and have studied corresponding sodium and potassium salts with carbonate (CO$_3$ $^{2-}$), acetate (CH$_3$COO$^{-}$), thiocyanate (SCN$^{-}$) as anions. The probable positions of the chosen ions in the Hofmeister series are displayed in Fig. 1 [22, 49]. The left end of the series corresponds to the more *“hydrophilic”* ions while at the right end the *“hydrophobic”* ions are located.

**Fig. 2:**
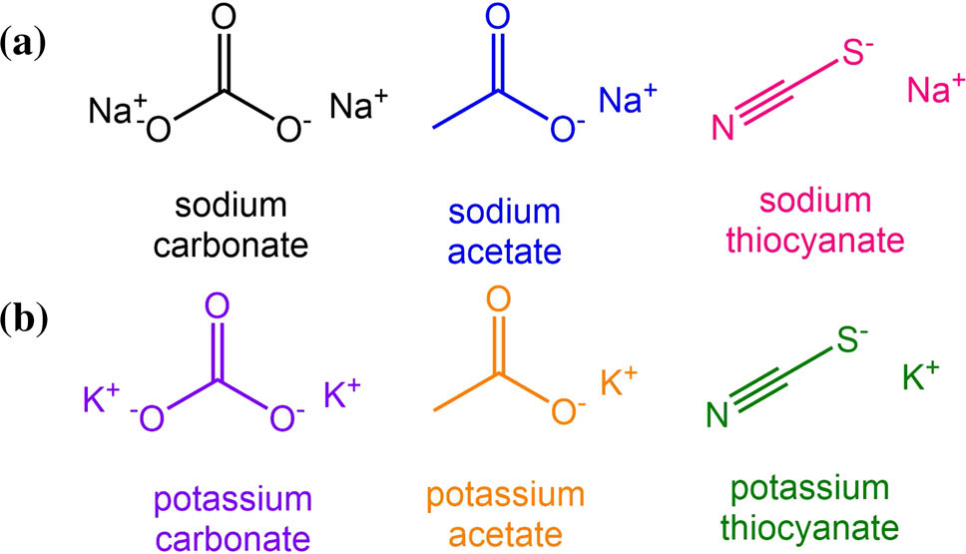
**a** Chemical structure of the investigated sodium salts and **b** the structure of the investigated corresponding potassium salts [15]. The color of the chemical structures corresponds to that of the symbols in the figures

The chemical structures of the investigated salt are shown in Fig. 2. For carbonate and acetate salts studied, we expect the thermodiffusive behavior to be dominated by hydrogen bonds as both the cations and anions are hydrophilic. For thiocyanate salts, the thermophoretic behavior will have contributions from ionic as well as hydrogen bonding effects. To investigate whether these hypotheses is in line with the results and how it depends on the cation, we systematically investigate these salts in a temperature range of 15 to 45$^\circ$C with concentration being varied from 0.5 to 5 mol kg$^{-1}$.

## Experimental section

### Sample preparation

Deionized water from a Millipore filter unit (0.22 $\mu$m) was used to prepare all aqueous solutions. Potassium carbonate (K$_2$CO$_3$), sodium carbonate (Na$_2$CO$_3$), potassium acetate(CH$_3$COOK), sodium acetate(CH$_3$COONa), potassium thiocayante (KSCN) and sodium thiocayante (NaSCN) were purchased from Sigma-Aldrich and used without further purification. The salts were of purity $\ge$ 99$\%$. Solutions ranging from concentration 0.5-5 mol kg$^{-1}$ were prepared using a stock solution at a high concentration.

An optical quartz cell (Hellma) with an optical path length of 0.2 mm were used for measurement of the thermophoretic properties using infrared thermal diffusion forced Rayleigh scattering (IR-TDFRS). Prepared solutions were filtered through a 0.2 $\mu$m filter (Whatman Anotop 10) and filled into this quartz cell. All measurements are performed in the temperature range between 15 and 45$^ \circ$C. Measurements were performed at least two times in different cells with freshly prepared samples. The experimental methods which are used to measure thermodiffusion are explained in detail in Supporting Information.

## Results

### Concentration dependence of $S_\mathrm {T}$ and $D_\mathrm {T}$

**Fig. 3:**
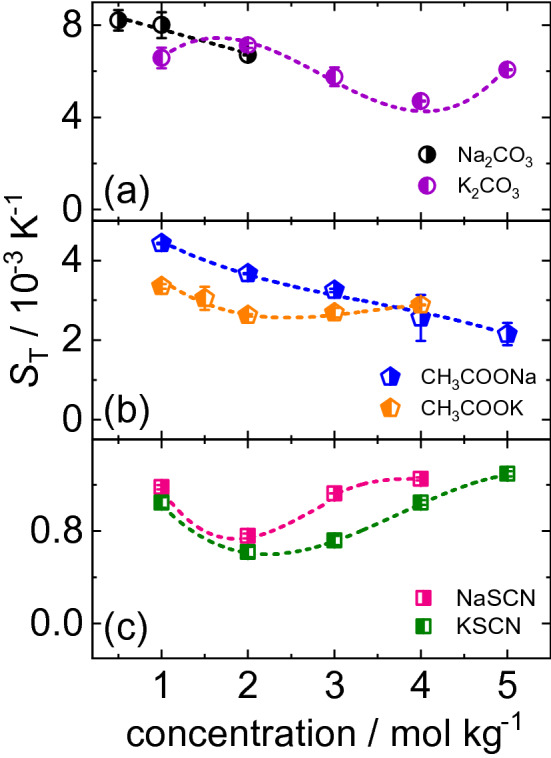
Soret coefficient of **a** K$_2$CO$_3$ (violet circles), Na$_2$CO$_3$ (black circles), **b** CH$_3$COOK (orange pentagons), CH$_3$COONa (blue pentagons), **c** KSCN (green squares) and NaSCN (pink squares) in water as a function of concentration at $T=25^{\circ }$C. The lines are fits according to Eq. 2. Further information is given in the text

The concentration dependence of $S_\mathrm {T}$ for all studied salt solutions is shown in Fig. 3. The lines in Fig. 3 correspond to the fit corresponding to Eq. (2). An example of the raw IR-TDFRS signal for NaSCN at $T=25^{\circ }$C is shown in the Supporting Information (Fig. S5). Except for Na$_2$CO$_3$, third order and second order polynomials have been used to describe the concentration and temperature dependence of $S_\mathrm {T}$, respectively. For Na$_2$CO$_3$ for which measurements were not possible above 2 mol kg$^{-1}$ due to its solubility limit, first order polynomials of concentration and temperature have been used to describe the data.

The overview Fig. 3 shows that as the hydrophilicity of the anion decreases, the magnitude of the Soret coefficient also decreases. The values for the two cations are very similar. It has be noted that at low concentrations ($\le$ 2 mol kg$^{-1}$), all sodium salts have a stronger tendency to accumulate on the cold side. All sodium salts have lower solubility than the corresponding potassium salts, with the difference being most pronounced for the two carbonate salts. $S_\mathrm {T}$ of the divalent salt K$_2$CO$_3$ oscillates, while $S_\mathrm {T}$ of the corresponding sodium salt decays monotonously with concentration in the accessible range. A previously reported $S_\mathrm {T}=8.21\times 10^{-3}$ K$^{-1}$ value of Na$_2$CO$_3$ at 0.5 [50] agrees well with our $S_\mathrm {T}$-value of $8.19\times 10^{-3}$ K$^{-1}$. CH$_3$COOK shows a shallow minimum in $S_\mathrm {T}$, while CH$_3$COONa decreases monotonously with concentration. Both thiocyanate salts show a minimum in $S_\mathrm {T}$ with concentration at 2 mol kg$^{-1}$. This minimum in $S_\mathrm {T}$ shown by KSCN, NaSCN and CH$_3$COOK has been previously reported for certain other salts like KCl, NaCl and LiCl [51, 52]. Although, several salts exhibit this behavior, the physical reason for this minimum is not yet clear [15].

The concentration dependence of $D_\mathrm {T}$ has a similar behavior to that of $S_\mathrm {T}$ for all systems studied. This is shown in the Supporting Information (Fig. S6).

### Temperature dependence $S_\mathrm {T}$ and $D_\mathrm {T}$

**Fig. 4:**
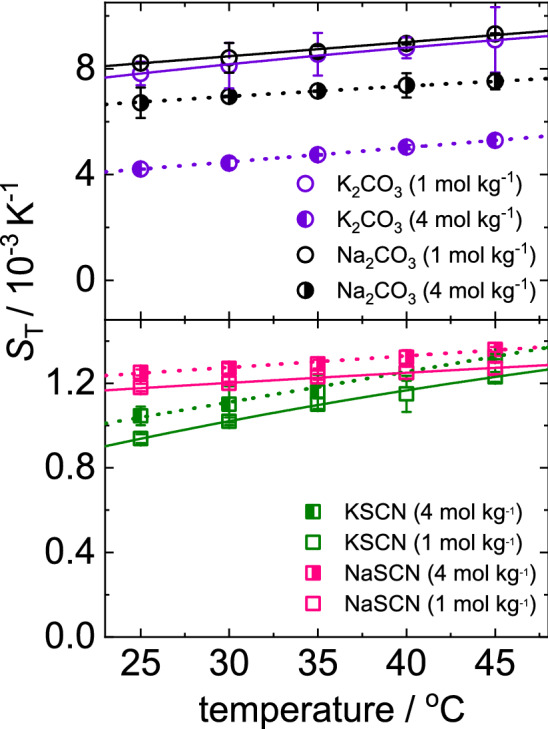
Soret coefficient of Na$_2$CO$_3$, K$_2$CO$_3$, NaSCN and KSCN as a function of temperature. Open and half-filled symbols correspond to concentrations 1 and 4 mol kg$^{-1}$ respectively. The half-filled symbols correspond to the symbols used in Fig. 3. The lines correspond to a fit according to Eq. 1

In the following we will discuss the temperature dependence of $S_\mathrm {T}$, which is for diluted solutes related to their hydrophilicity. Figure 4 shows the temperature dependence of $S_\mathrm {T}$ of four salts (Na$_2$CO$_3$, K$_2$CO$_3$, NaSCN and KSCN) at two concentrations (1 and 4 mol kg$^{-1}$). The line is a fit according to Eq. (1). In general, $S_\mathrm {T}$ shows an increase with temperature indicating system get more thermophobic with increasing temperature. For carbonates (K$_2$CO$_3$ and Na$_2$CO$_3$), $S_\mathrm {T}$ decreases with increasing concentration, while for thiocayanate salts(KSCN and NaSCN), magnitude of $S_\mathrm {T}$ increases with concentration. Acetate salts (CH$_3$COOK and CH$_3$COONa), exhibits behavior similar to carbonate salts which is shown in Supporting Information (cf. Fig. S1).

Although, for non-ionic solute molecules and many bio molecules the temperature sensitivity, $\Delta S_{\mathrm {T}}(\Delta T)$ = $S_\mathrm {T}(T+\Delta T)$-$S_\mathrm {T}(T)$ decreases with increasing temperature, while $\Delta S_{\mathrm {T}}(\Delta T)$ barely changes for salts. The temperature dependence of $S_{\mathrm {T}}$ of ionic solutes is different from the behavior of non-ionic solutes which has been studied before [6]. For non-ionic solutes only the most hydrophilic solutes exhibited at very low concentrations a temperature dependence of $S_{\mathrm {T}}$ described by Eq. 1 [6], whereas $S_{\mathrm {T}}$ of all investigated salts can be described for all concentrations by Eq. 1. This difference in behavior could be an effect of cluster formation at higher concentrations [19]. Those formed clusters are well hydrated thus behaving like diluted solutions of clusters. Additionally, the temperature sensitivity, $\Delta S_{\mathrm {T}}(\Delta T)$ = $S_\mathrm {T}(T+\Delta T)$-$S_\mathrm {T}(T)$ decreases for non-ionic solutes with increasing temperature, while this slope barely changes for the salts, which might be related to the fact that ionic interactions show a weaker temperature dependence than hydrogen bonds.

Due to decrease of the viscosity with increasing temperature, we expect that the diffusion coefficient *D* increases with temperature. This is observed for all the studied salts. As in the case of concentration dependence, $D_\mathrm {T}$ shows a similar trend as $S_\mathrm {T}$. For further information, see Supporting Information (cf. Fig. S7).

**Fig. 5:**
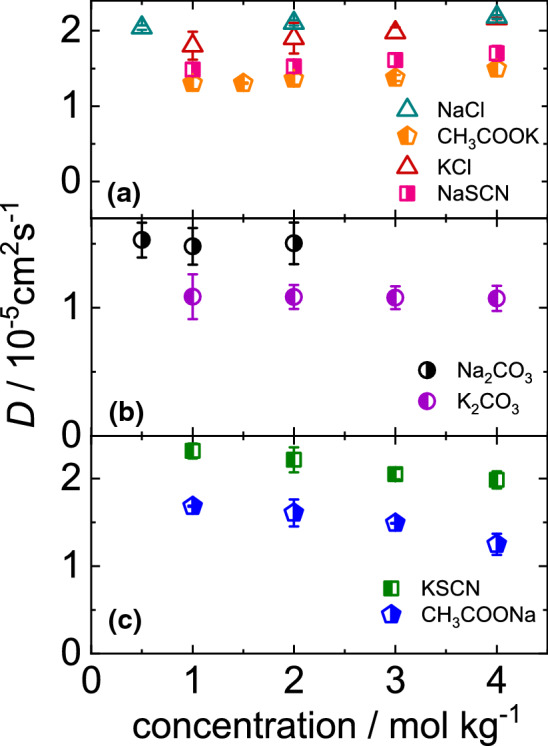
**a** Diffusion coefficient of **a** CH$_3$COOK (orange left-half-filled pentagons), NaSCN (pink right-half-filled squares) and Na$_2$CO$_3$ (black right-half-filled circles) as function of concentration at $T=25^\circ$C. For comparison we show also the literature values of sodium chloride (NaCl) (green open triangle down) [53] and potassium chloride (KCl) (red open triangle up) [15]. **b** Diffusion coefficient of aqueous Na$_2$CO$_3$ (black right-half-filled circles) and K$_2$CO$_3$ (violet left-half-filled circles) solutions as function of concentration at $T=25^\circ$C. **c** Diffusion coefficient of KSCN (green left-half-filled squares) and CH$_3$COONa (blue right-half-filled pentagons) as function of concentration at $T=25^\circ$C. Symbols correspond to the symbol used for $S_{\mathrm {T}}$ of the same salt in Fig. 3

### Concentration dependence of *D*

The dependence of the diffusion coefficient *D* on concentration at $25^{\circ } C$ is shown in Fig. 5. The studied salts can be classified in three groups. The first group (cf. Fig. 5a) shows a slight increase of *D*, in the second group (cf. Fig. 5b) *D* is independent of concentration and the third group (cf. Fig. 5c) shows a decrease of *D* with concentration. In the investigated concentration regime none of the salts shows a clear minimum as predicted by theories [42]. The diffusion coefficient of the third group with KSCN and CH$_3$COONa decays with concentration. From molecular dynamic simulations it is known that both salts form network structures, which are interlinked with the water network slowing down the diffusion at higher concentrations [19, 46].

## Discussion

### Concentration and temperature dependence of $S_\mathrm {T}$

For comparison of the concentration dependence of $S_\mathrm {T}$ for the various salt systems we introduce $\Delta S_{\mathrm {T}}(\Delta c)$ as $\Delta S_{\mathrm {T}}(\Delta c) = S_\mathrm {T}(2~\mathrm {mol~kg}^{-1}) - S_\mathrm {T}(1~\mathrm {mol~kg}^{-1})$. The concentration dependence of the salt systems studied indicates that, with the exception of CH$_3$COONa, all salts show an increase in thermophobicity at higher concentrations. For CH$_3$COONa, the thermophobicity decreases monotonously with increasing concentration. A decay of the Soret coefficient with increasing concentration ($\Delta S_{\mathrm {T}}(\Delta c)<0$) has also been observed for ethanol [27, 54], for acetamide and *N*-methyl-formamide in water [6]. In all systems, $\Delta S_{\mathrm {T}}(\Delta T)$ increases as a function of concentration, resulting in a temperature-independent intersection point ($\Delta S_{\mathrm {T}}(\Delta T)=0$), if we plot $S_{\mathrm {T}}$ versus concentration.

Except for K$_2$CO$_3$ and NaSCN, $\Delta S_{\mathrm {T}}(\Delta c)$ is nearly independent of temperature (see also supporting information Figs. S2, S3 and S4). For K$_2$CO$_3$, $\Delta S_{\mathrm {T}}(\Delta c)$ shows a weak increase with temperature, while for KSCN it shows a decrease with temperature. The physical origin of this behavior is not yet clear. While the change in the concentration dependence of $S_{\mathrm {T}}$ for different temperatures of non-ionic solutes can be related to the fact that the thermophoretic behavior of these systems is determined exclusively by hydrogen bonds, which show a strong temperature dependence, the electrostatic interactions are less temperature dependent.

**Fig. 6:**
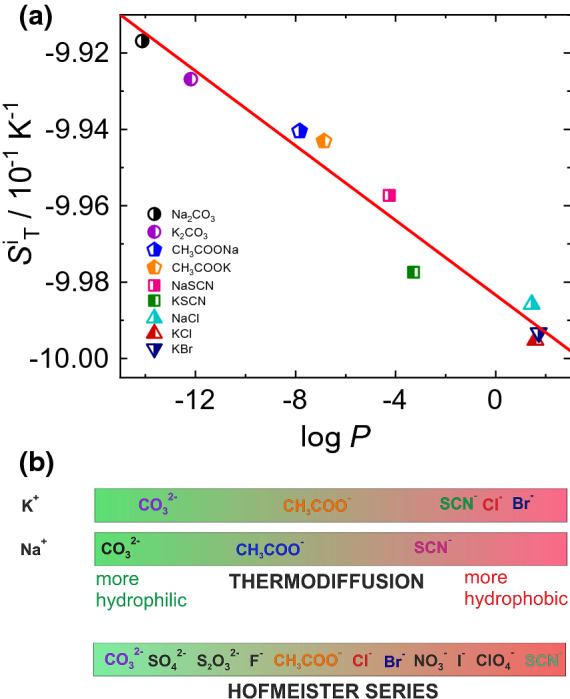
**a** $S_\mathrm {T}^i$ values of all studied systems plotted as function of $\log P$. Note, that $\log P$ is the sum of an ionic and non-ionic contribution. The $S_\mathrm {T}^i$ values of KCl and KBr have been previously reported [15]. $S_\mathrm {T}^i$ of NaCl was obtained by fitting the $S_\mathrm {T}$ values reported by Wang *et al.* [53]. First order polynomials of concentration and temperature have been used to fit the data using Eq.2 for NaCl. **b** Sequence of the anions based on $S_\mathrm {T}^i$ for the two investigated cations in comparison with the Hofmeister series

We used Eq. 2 to describe the concentration and temperature dependence of $S_\mathrm {T}$ and determined the parameter $S_\mathrm {T}^i$ for all salt systems studied. We set $a_0=0$ as it is strongly coupled to $S_{\mathrm T}^i$. Note, that the observed correlation between $S_{\mathrm T}^i$ and $\log P$ remains, when $a_0$ is fixed. Figure 6 shows that $S_\mathrm {T}^i$ depends linearly on $\log P$. The most hydrophilic salt, Na$_2$CO$_3$ has the highest $S_\mathrm {T}^i$-value and KBr, the most hydrophobic salt has the lowest $S_\mathrm {T}^i$. Both investigated cations potassium and sodium follow the same overall trend and show no systematic deviations. Note, that $S_\mathrm {T}^i$ of non-polar systems is correlated with the difference in mass and moment of inertia of the two compounds [2]. The correlation between $S_\mathrm {T}^i$ and $\log P$ holds apparently for ionic as well as for non-ionic water soluble solutes and is most probably related to ability of molecules to form hydrogen bonds, but so far there is no microscopic theory [15]. It should also be noted that the correlation is likely to be limited to molecules small enough that they do not coil or fold, so that the entire surface of the molecule is accessible to the solvent.

As already discussed in Sect. 1, Hofmeister series arranges ions on the basis of their decreasing hydrophilicity [23]. log *P* is the parameter that defines the hydrophilicity of a solute molecule. A negative value of log *P* indicates that the molecule has a higher affinity towards aqueous phase (hydrophilic in nature). On the other hand, a positive value denotes a higher concentration in the organic phase (hydrophobic in nature) [55, 56]. Based on the $\log P$-dependence of $S_\mathrm {T}^i$ we can define a new hydrophobic-hydrophilic scale for the anions displayed in Fig. 6b. The anions CO$_3^{2-}$, CH$_3$COO$^-$ and SCN$^-$ have independent of the cation the same order as in the Hofmeister Series, while Cl$^-$ and Br$^-$ are more hydrophobic than SCN$^{-}$ according to their thermodiffusive behavior. Note that, corresponding to the $\log P$-scale Na$^+$ is slightly more hydrophilic than K$^+$, which is reversed in the Hofmeister series. This difference is not completely understood, but might be related to the fact that Hofmeister ranks individual ions and not complete salts.

**Fig. 7:**
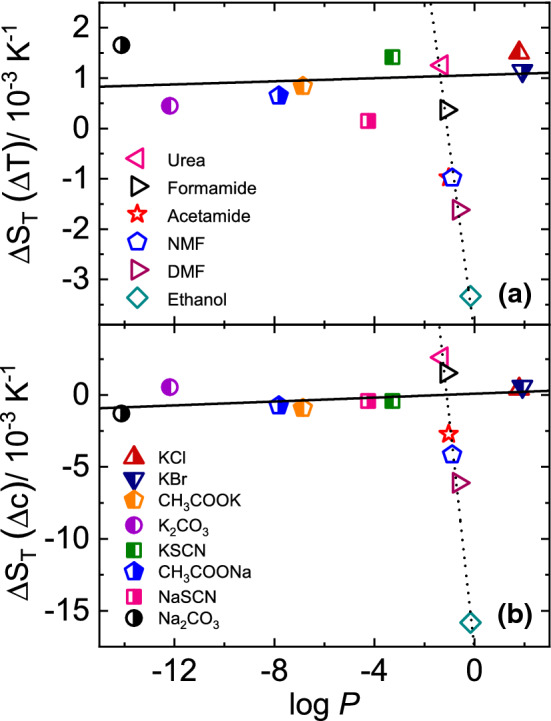
**a** Change of $\Delta S_{\mathrm {T}}(\Delta T)$ and **b** change of $\Delta S_{\mathrm {T}}(\Delta c)$ as function of $\log P$ for the investigated salt systems in comparison with some previously studied non-ionic systems [6]. The solid and dotted lines are linear fits for ionic and non-ionic systems, respectively. Further information is given in the text

For non-ionic systems it is known that changes of the Soret coefficient $\Delta S_{\mathrm {T}}(\Delta T)$ and $\Delta S_{\mathrm {T}}(\Delta c)$ in a certain temperature $\Delta T$ and concentration $\Delta c$ interval correlate with $\log P$. For the investigated salts we define $\Delta S_{\mathrm {T}}(\Delta T)= S_{\mathrm {T}}(50^\circ \mathrm {C})-S_{\mathrm {T}}(20^\circ \mathrm {C})$ at 1 mol kg$^{-1}$ and $\Delta S_{\mathrm {T}}(\Delta c)$ as mentioned before in Sect. 4.1 at $T=25^{\circ }$C. Figure 7 displays $\Delta S_{\mathrm {T}}(\Delta T)$ and $\Delta S_{\mathrm {T}}(\Delta c)$ as function of $\log P$. For comparison we show also the results for the previously investigated non-ionic systems [6]. The solid and dotted lines are linear fits for ionic and non-ionic systems, respectively. Note, that for non-ionic solutes $\Delta S_{\mathrm {T}}(\Delta T)= S_{\mathrm {T}}(50^\circ \mathrm {C})-S_{\mathrm {T}}(20^\circ \mathrm {C})$ at 5wt% and $\Delta S_{\mathrm {T}}(\Delta c)= S_\mathrm {T}(50\mathrm {wt}\%)-S_\mathrm {T}(5\mathrm {wt}\%)$ at $T=10^{\circ }$C. Neither $\Delta S_{\mathrm {T}}(\Delta T)$ nor $\Delta S_{\mathrm {T}}(\Delta c)$ shows a pronounced $\log P$-dependence as observed for the non-ionic solutes. Thus, $\Delta S_{\mathrm {T}}(\Delta T)$ and $\Delta S_{\mathrm {T}}(\Delta c)$ of the salts are nearly independent of the hydrophilicity of the salt, in clear contrast to non-ionic solutes, which are very sensitive to a change in $\log P$. Hydrophilic, non-ionic solutes form more hydrogen bonds with water, the number of which decreases when the temperature or concentration is increased. This then also leads to a strong decrease of $\Delta S_{\mathrm {T}}(\Delta T)$ and $\Delta S_{\mathrm {T}}(\Delta c)$ with increasing $\log P$. For ionic solutes $\Delta S_{\mathrm {T}}(\Delta T)$ and $\Delta S_{\mathrm {T}}(\Delta c)$ are more or less independent of $\log P$. Therefore, we assume that the thermophoretic behavior ionic solutes at the first order are determined by electrostatic interactions, which are independent of the hydrophilicity. Further studies are required to investigate whether the thermophoretic behavior shows some correlation with $\log P$ at much lower concentrations or for salts with larger organic side groups.

### Concentration dependence of *D*

**Fig. 8:**
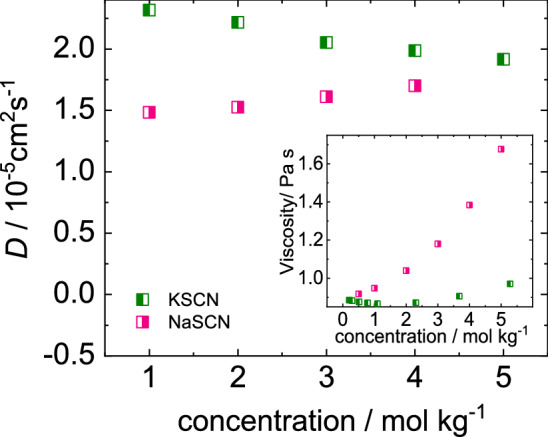
Change of diffusion coefficient *D* and viscosity of thiocyanate salts studied with concentration at a temperature of $25^{\circ }$C

The measurement of the diffusion coefficient gives information about the entities and their interactions diffusing in the temperature gradient (cf. Sect. 3). Figure 8 clearly demonstrates that changing the cation can have a noticeable effect on the diffusion coefficient of salts. It shows that the diffusion coefficient of sodium thiocyanate in water at 25$^\circ$C increases with concentration, while that of the corresponding potassium salts decreases. Differences are also observed for aqueous solutions of the acetate-anions, but in this case the diffusion coefficient of the sodium salt in water decreases, while the corresponding potassium salt shows a slight increase with concentration (cf. Fig. 5a and c). The decrease of the diffusion coefficient might be related to the stronger association of Na$^{+}$ compared to K$^{+}$ in the presence of the acetate anion [47]. For aqueous KSCN solutions the formation of network structures [19, 46] as well as the formation of clusters have been reported [35]. For both scenarios a decrease of the diffusion coefficient with increasing concentration is expected.

The measured diffusion coefficient depends inversely on the viscosity of the solutions. In the inset of Fig. 8 literature values of the viscosity of both thiocyanate salts are displayed [57]. It is obvious that the change of the cation has also a prominent effect on the viscosity. Considering the viscosity we would expect that the diffusion of NaSCN should show a more pronounced decrease with increasing concentration compared to KSCN. This is obviously not the case, therefore we looked also into the differences in the chemical potential of the two salt solutions. According to Eq. 4 the diffusion coefficient depends on the mean activity coefficient. Comparing the term $1+c({d \ln \gamma _\pm }/{dc})$ for NaSCN and KSCN solutions, we find that the increase of the term for NaSCN is four times larger than for KSCN (cf. Supporting Information Sect. S7). This might be the reason that we observe a weak increase of *D* for NaSCN with concentration although a recent work predicts for both salts cluster formation with increasing concentration [35]. Note, that the clustering for NaSCN is less pronounced, as the percentage of clustered ions at 4 mol/kg is 60% and 67% for NaSCN and KSCN, respectively. Therefore, the cluster formation dominates the diffusion only for KSCN.

## Conclusion

We have investigated the thermophoretic properties of various aqueous sodium salt solutions as function of temperature and concentration and compared the results with those measured for the corresponding potassium salts to explore the influence of the cation exchange.

It turned out that the temperature and concentration dependence of the Soret coefficient is only marginally influenced by the exchange of the cation (cf. Figs. 3 and 4). The shape of the curves is similar although not identical. The diffusion coefficient *D* is influenced by the exchange of the cation. While *D* of CH$_3$COOK and NaSCN in water shows a weak increase with concentration the corresponding salts CH$_3$COONa and KSCN show a decay (cf. Fig. 5a and c). For both divalent salts we observe no dependence on the concentration. Therefore, we can only conclude that the exchange of cation can influence the behavior, but so far we were not able to identify a trend. And the influence is especially visible in the diffusion, but not so much in the thermophoretic behavior.

As already observed for the studied potassium salts the temperature dependence of the Soret coefficient can be described for all concentration with Eq. 1. While for non-ionic systems the $\Delta S_{\mathrm {T}}(\Delta T)$ changes with concentration, the ionic systems show only a shift of $S_{\mathrm {T}}$. This is also the reason that in contrast to the non-ionic systems $\Delta S_{\mathrm {T}}(\Delta c)$ and $\Delta S_{\mathrm {T}}(\Delta T)$ are almost independent of $\log P$, while for non-ionic systems a strong dependence is found (cf. Fig. 7).

Further we were able to describe the temperature and concentration dependence of $S_{\mathrm {T}}$ using Eq. 2 and observed that the parameter $S_\mathrm {T}^i$ shows a linear correlation with $\log P$. The here investigated anions have the same sequence as in the Hofmeister series. Deviations are found for Cl$^-$ and Br$^-$. The sequence of the two investigated cations Na$^+$ and K$^+$ is reversed. But as the cations lie very close together within the Hofmeister Series, more experiments need to be performed covering the entire range.

Our studies, thus explore the effect of both cations and anions on the thermophoretic properties of salt systems, which play a crucial role in science and technology [8, 11, 14]. In this study we have chosen rather simple experimental conditions by looking into aqueous salt solutions. In order to learn more about the role of the thermodiffusion of ions and the coupling of thermal and electric fields it would also be desirable to perform studies in biological cells or thermoelectric devices. This is still a big challenge for future studies.

## Supplementary Information

Below is the link to the electronic supplementary material.

Supplementary file 1 (pdf 834 KB)
