# Supplementary material for: Towards understanding specific ion effects in aqueous media using thermodiffusion
Source: Eur Phys J E Soft Matter. 2022 Feb 1;45(2):10. doi: 10.1140/epje/s10189-022-00164-8 (PMC8807466; doi:10.1140/epje/s10189-022-00164-8)
Supplement: Supplementary file 1 — (pdf 834 KB) [file 10189_2022_164_MOESM1_ESM.pdf]

SUPPORTING INFORMATION: Effect of cation on the thermodiffusive behavior of aqueous salts solutions

Shilpa Mohanakumar
and Simone Wiegand

Contents

S1 Experimental Methods **S2**
S1.1 Thermal Diffusion Forced Rayleigh Scattering S2
S1.2 Refractive index contrast measurements S2

S2 Temperature dependence of S_T for CH_3COOK and CH_3COONa **S3**

S3 Concentration dependence of S_T **S3**

S4 TDFRS signal **S6**

S5 Determined and used parameters **S6**
S5.1 Determined parameters: S_{T}^{i} and b_1 . . . . S6
S5.2 Used $\log P$-values S7

S6 Concentration and temperature dependence of D_T **S9**

S7 Concentration dependence of activity coefficient **S11**

S1 Experimental Methods

S1.1 Thermal Diffusion Forced Rayleigh Scattering

Thermodiffusion of the electrolyte solutions was measured by infrared thermal diffusion forced Rayleigh scattering (IR-TDFRS) [1, 2]. This method uses the interference grating of two infrared laser beams (λ = 980 nm) to generate a temperature grating inside an aqueous sample due to the inherent absorbtion of water in that range [3]. A third laser beam is refracted by this grating and the intensity of the refracted beam is measured. This intensity is proportional to the refractive index contrast of the grating, showing a fast rise over time due to the thermal gradient, then a slower change of intensity due to diffusion of the solute along the temperature gradient (cf. Fig 1).

The heterodyne scattering intensity $\zeta_{het}(t)$ of the read-out beam is measured and fitted with

$$\zeta_{\mathrm{het}}(t) = 1 - \exp\left(-\frac{t}{\tau_{\mathrm{th}}}\right) - A\left(\tau - \tau_{\mathrm{th}}\right)^{-1} \times \left\{\tau\left[1 - \exp\left(-\frac{t}{\tau}\right)\right] - \tau_{\mathrm{th}}\left[1 - \exp\left(-\frac{t}{\tau_{\mathrm{th}}}\right)\right]\right\}. \tag{S1}$$

With the lifetimes $\tau_{th} = (D_{th}q^2)^{(-1)}$ and $\tau = (Dq^2)^{(-1)}$ of the temperature and concentration grating, respectively, where q, D_{th} and D denote the grating wave vector, the thermal diffusivity and the mutual diffusion coefficient, respectively. The Soret coefficient(S_{T}) can be calculated from the amplitude A, if the so-called contrast factors, the change of refractive index with temperature and concentration, $(\partial n/\partial T)_{c,p}$ and $(\partial n/\partial c)_{T,p}$, are known:

$$A = \left(\frac{\partial n}{\partial c}\right)_{p,T}\left(\frac{\partial n}{\partial T}\right)_{p,c}^{-1} S_{\mathrm{T}}c\,(1-c)\,. \tag{S2}$$

S1.2 Refractive index contrast measurements

Refractive index contract factors are required to calculate S_{T}. The refractive index as function of concentration was measured with an Abbe refractometer (Anton Paar Abbemat MW) at a wavelength of 632.8 nm. For all salts, refractive index at five concentrations around the desired contraction were measured. The slope of the linear interpolation of the refractive index as a function of concentration gives $(\partial n/\partial c)_{p,T}$. The refractive index increments with temperature $(\partial n/\partial T)_{p,c}$ was measured interferometrically [4]. Measurements were performed over a temperature range of 25-45°C, with a heating rate of 1.6 mK/sec. The refractive index varied linearly with concentration and temperature in the investigated range.

S2 Temperature dependence of S_T for CH_3COOK and CH_3COONa

As mentioned in the main manuscript, temperature dependence of S_T of CH_3COOK and CH_3COONa is similar to that of carbonate salts. This is shown in Fig.S1.

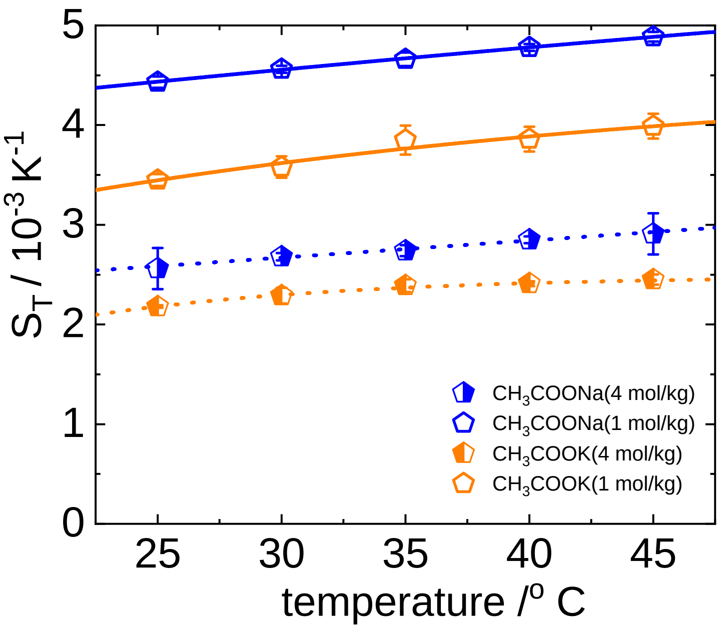

Figure S1: Soret coefficient of CH_3COOK and CH_3COONa as a function of temperature. Open and half-filled symbols correspond to concentrations 1 and 4 mol kg^{-1}, respectively. The lines correspond to fit according to Eq. (1) in the main manuscript.

S3 Concentration dependence of S_T

The concentration dependence of different salt systems studied at three different temperatures (25°C, 35°C, 45°C) is shown in Figs.S2, S3 and S4. It can be seen that the concentration dependent slope of all the studied systems doesn't change vastly with temperature.

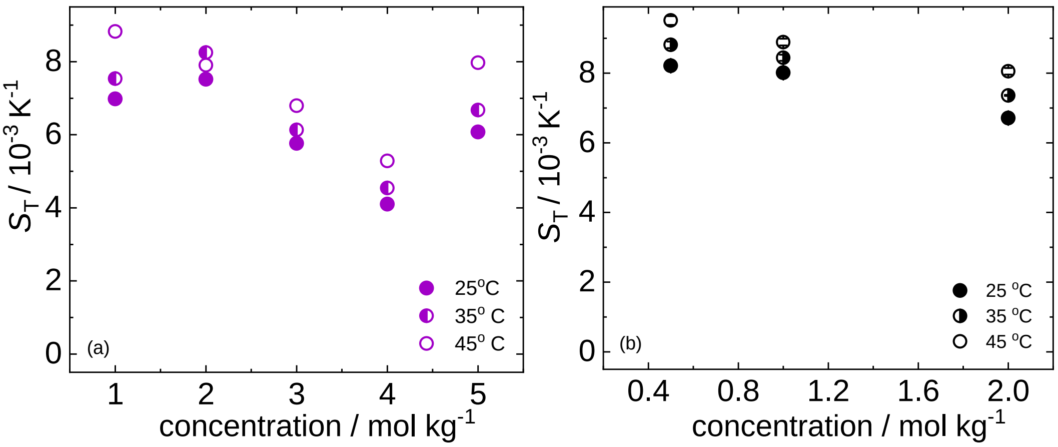

Figure S2: Concentration dependence of S_T of (a)K_2CO_3 and (a)Na_2CO_3 at 25° C, 35° C and 45° C

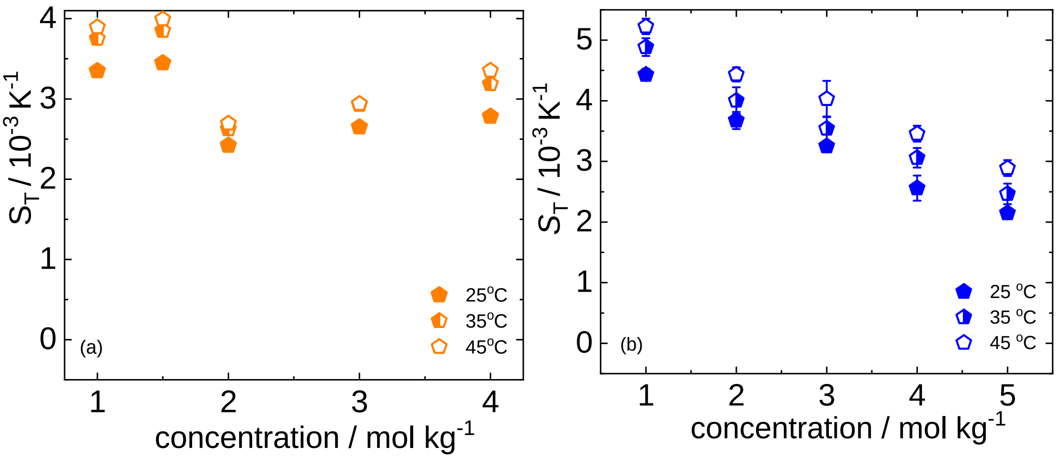

Figure S3: Concentration dependence of S_T of (a)CH_3COOK and (a)CH_3COONa at 25° C, 35° C and 45° C

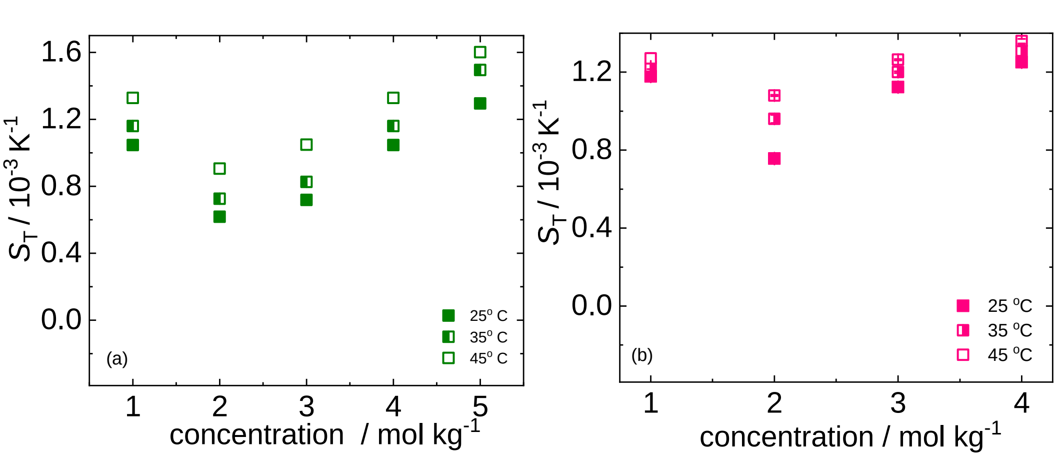

Figure S4: Concentration dependence of S_{T} of (a)KSCN and (a)NaSCN at 25° C, 35° C and 45° C

S4 TDFRS signal

An example of the TDFRS raw-data is shown in Fig.S5. Fig.S5(a) and Fig.S5(b) corresponds to the signal measured for NaSCN, at 1 mol kg^{-1} and 4 mol kg^{-1} respectively at 25° C and the corresponding residual plots. The black dots marks the data points, the red line represents the fit according the Eq.S2 and the red dots mark the residuals, which are within the 2σ range and do not show systematic deviations.

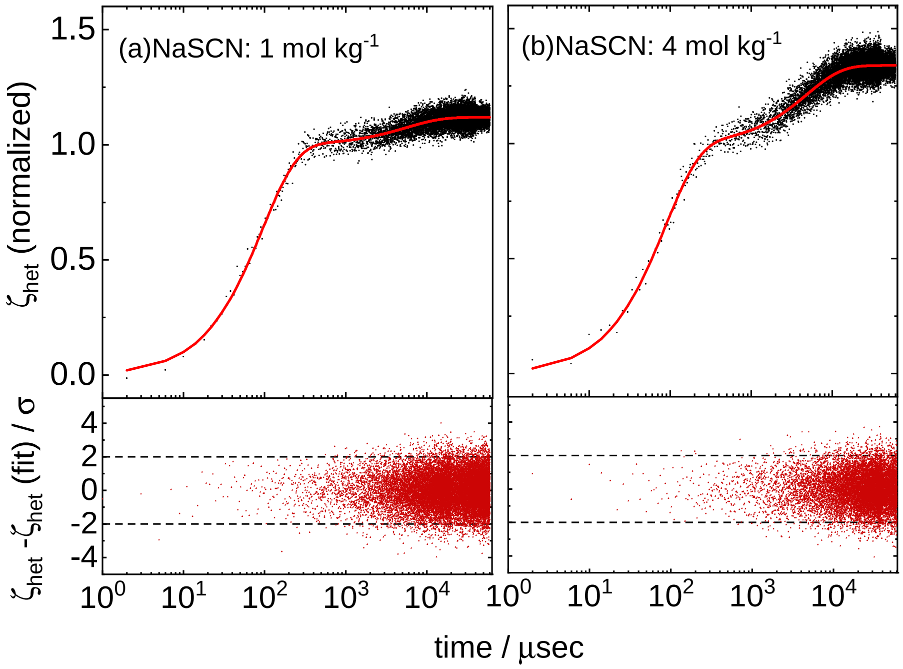

Figure S5: Normalized heterodyne diffraction intensities of NaSCN at 25°C as a function of time at (a) 1 mol kg^{-1} and (b) 4 mol kg^{-1}. The black dots marks the data points, the red line represents the fit according the Eq.S2 and the red dots mark the residuals.

S5 Determined and used parameters

S5.1 Determined parameters: S_{T}^{i} and b_1

We described the temperature and concentration dependence of the Soret coefficient of the investigated salt solutions successfully using Eq. (2) in the main manuscript. The determined S_{T}^{i} values are listed in Table S1. It has to be noted here that for all salts except Na_2CO_3 the fitting corresponds to

third order and second order polynomials for concentration and temperature respectively. Due to the low solubility of Na_2CO_3, we were only able to measure the three lowest concentration, therefore we had to reduce the number of fit parameters by using first order polynomials of concentration and temperature to describe the data. For NaCl as well, for which the S_T values were only reported at three concentrations[5], we used first order polynomials of concentration and temperature to fit the data.

Table S1: S_T^i values of investigated systems

	S_T^i ($\times 10^{-1}$K^{-1})	b_1(K^{-1})
CH_3COONa	9.94±0.3	12.7±0.6
CH_3COOK	9.94±0.1	13.6±0.1
Na_2CO_3	9.92±0.4	19.7±0.1
K_2CO_3	9.93±0.1	19.6±0.5
NaSCN	9.96±0.2	3.5±0.9
KSCN	9.97±0.3	3.3±0.3
NaCl	9.98±0.2	2.9±0.4
KCl	9.99±0.3	1.0±0.7
KBr	9.99±0.1	4.0±0.2

S5.2 Used $\log P$-values

In order to correlate S_T^i with the hydrophilicity, we also determined the log P. Calculator Plugins were used for calculation of $\log P$ within Marvin 16.5.2.0, 2016, ChemAxon (http://www.chemaxon.com). The calculation method is based on the publication by Viswanadhan *et al.* [6]. Note that different methods exists, which give slightly different values and it is important to stay in one method to compare results. Values obtained are listed in Table S2. It has to be noted that $\log P$ has ionic and non-ionic contributions and the values listed here corresponds to the sum of these contributions.

Table S2: log P values of investigated systems

	log P
CH_3COONa	-7.83
CH_3COOK	-6.86
Na_2CO_3	-14.12
K_2CO_3	-12.18
NaSCN	-4.25
KSCN	-3.28
NaCl	1.44
KCl	1.59
KBr	1.72

S6 Concentration and temperature dependence of D_T

Dependence of D_T with concentration at 25° C is shown in Fig.S6. The behavior of D_T is similar to that of S_T, which has been discussed in the main manuscript in Sec. 3.

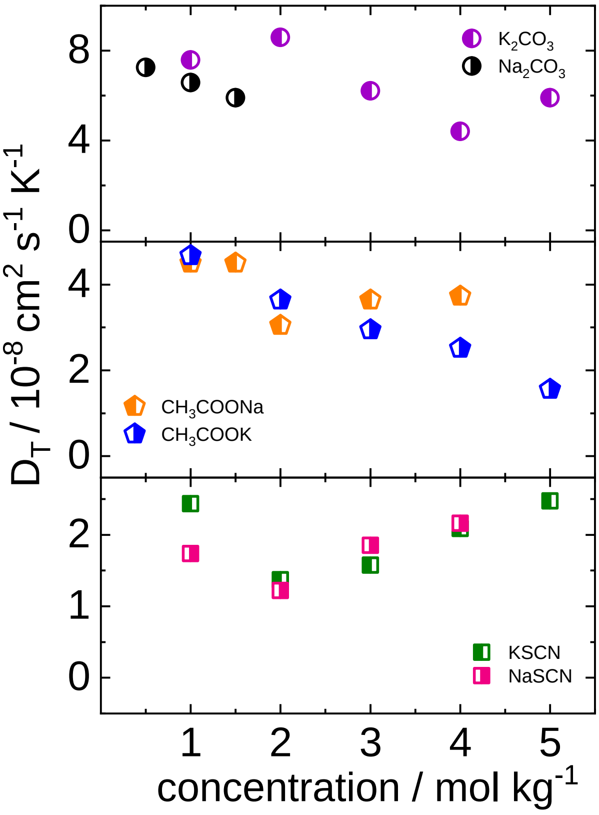

Figure S6: Thermal diffusion coefficient of all investigated salts as a function of concentration at a temperature of 25°C. We used the symbols as follows: CH_3COOK(orange pentagons), CH_3COONa(blue pentagons), K_2CO_3(violet circles), Na_2CO_3(black circles), KSCN(green squares) and NaSCN(pink squares).

Temperature dependence of D_T at the lowest concentration measured (1 mol kg^{-1}) is shown in Fig.S7.

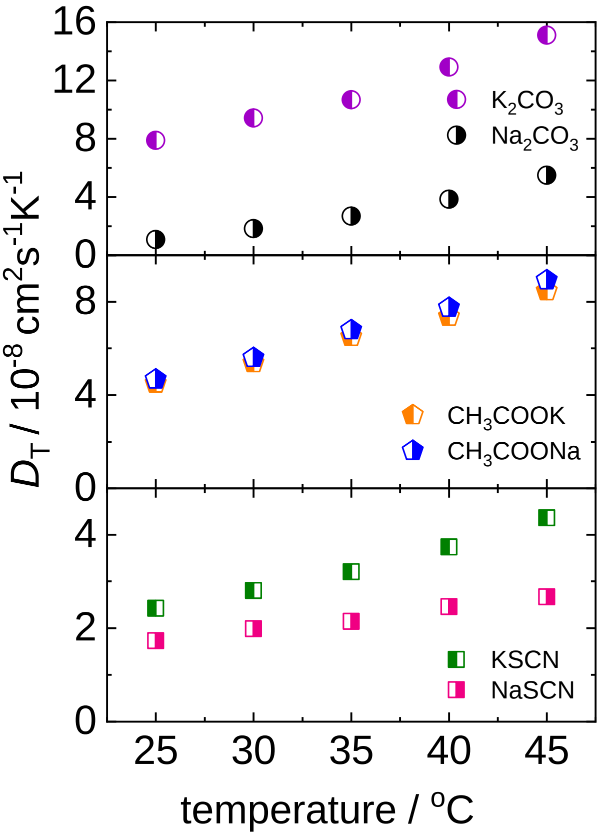

Figure S7: Thermal diffusion coefficient of all investigated salts as a function of temperature at a concentration of 1 mol kg^{-1}. We used the symbols as follows: CH_3COOK(orange pentagons), CH_3COONa(blue pentagons), K_2CO_3(violet circles), Na_2CO_3(black circles), KSCN(green squares) and NaSCN(pink squares).

S7 Concentration dependence of activity coefficient

As discussed in the main manuscript (cf. Eq.4), D of electrolyte solutions depends on the mean ionic activity coefficient, $\gamma_{\pm}$. The concentration dependence of D for aqueous solutions of KSCN and NaSCN shows an decrease and increase, respectively. Both systems show an increase of the viscosity with increasing concentration. To understand the difference in the concentration dependence in the diffusion of the two salts, we examined additionally the concentration dependence of the mean ionic activity coefficient $\gamma_{\pm}$, which has been studied by Robinson *et al.* [7]. We have used these values to calculate the corresponding term $1+c(d\ln\gamma_{\pm}/dc)$ in Eq.4 in the main manuscript. This term shows for both salts a minimum at a low concentration around 0.25 mol/Kg and a gradual increase with increasing concentration. Within the measured concentration range (1-4 mol kg^{-1}), the increase of $1+c(d\ln\gamma_{\pm}/dc)$ is 4-times steeper for NaSCN compared to KSCN.

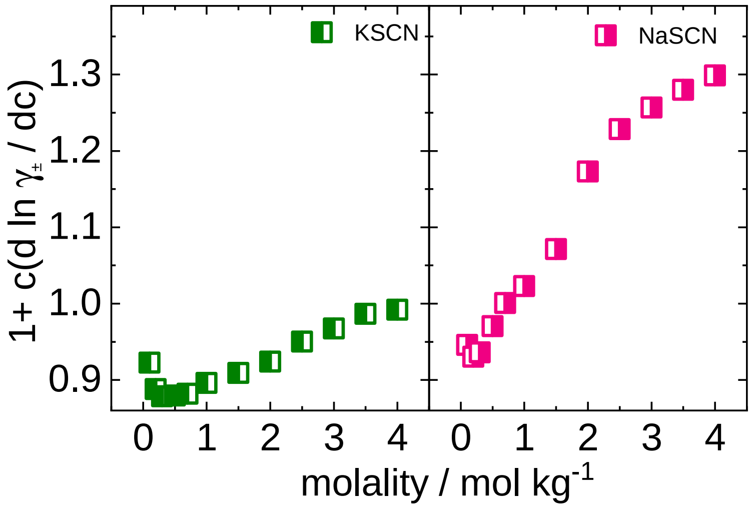

Figure S8: Concentration dependence of $1 + c(d\ln\gamma_{\pm}/dc)$ of NaSCN and KSCN.

References

[1] S. Wiegand and W. Köhler. Measurement of transport coefficients by an optical grating technique. *LNP Vol. 584: Thermal Nonequilibrium Phenomena in Fluid Mixtures*, 584:189–210, 2002.

[2] P. Blanco, H. Kriegs, M. P. Lettinga, P. Holmqvist, and S. Wiegand. Thermal diffusion of a stiff rod-like mutant y21m fd-virus. *Biomacromolecules*, 12:1602–1609, 2011.

[3] S. Wiegand, H. Ning, and H. Kriegs. Thermal diffusion forced rayleigh scattering setup optimized for aqueous mixtures. *J. Phys. Chem. B*, 111:14169–14174, 2007.

[4] A. Becker, W. Köhler, and B. Müller. A scanning michelson interferometer for the measurement of the concentration and temperature derivative of the refractive- index of liquids. *Phys. Chem. Chem. Phys.*, 99:600–608, 1995.

[5] F. Römer, Z. Wang, S. Wiegand, and F. Bresme. Alkali halide solutions under thermal gradients: Soret coefficients and heat transfer mechanisms. *J. Phys. Chem. B*, 117:8209–8222, 2013.

[6] Vellarkad N. Viswanadhan, Arup K. Ghose, Ganapathi R. Revankar, and Roland K. Robins. Atomic physicochemical parameters for three dimensional structure directed quantitative structure-activity relationships. 4. additional parameters for hydrophobic and dispersive interactions and their application for an automated superposition of certain naturally occurring nucleoside antibiotics. *J. Chem. Inf. Model.*, 29:163–172, 1989.

[7] R. A. Robinson. The activity coefficients of sodium and potassium thiocyanate in aqueous solution at 25 degrees from isopiestic vapor pressure measurements. *J. Am. Chem. Soc.*, 62:3131–3132, 1940.
